# Metabolomic Classification of Myalgic Encephalomyelitis/Chronic Fatigue Syndrome via Explainable Ensemble Learning and Pareto-Guided Feature Selection

**DOI:** 10.3390/ijms27135920

**Published:** 2026-06-30

**Authors:** Fatma Hilal Yagin, Yavuz Korkmaz, Cemil Colak, Sarah A. Alzakari, Amal K. Alkhalifa, Fahaid Al-Hashem, Mohammadreza Aghaei

**Affiliations:** 1 Department of Biostatistics, Faculty of Medicine, Malatya Turgut Ozal University, Malatya 44210, Türkiye; 2 Department of Family Medicine, Faculty of Medicine, Malatya Turgut Ozal University, Malatya 44210, Türkiye; 3 Department of Biostatistics and Medical Informatics, Faculty of Medicine, Inonu University, Malatya 44280, Türkiye; 4 Department of Computer Sciences, College of Computer and Information Sciences, Princess Nourah bint Abdulrahman University, P.O. Box 84428, Riyadh 11671, Saudi Arabia; 5 Department of Physiology, College of Medicine, King Khalid University, Abha 61421, Saudi Arabia; 6 Department of Ocean Operations and Civil Engineering, Norwegian University of Science and Technology (NTNU), 6009 Ålesund, Norway; 7 Department of Sustainable Systems Engineering (INATECH), Albert Ludwigs University of Freiburg, 79110 Freiburg, Germany

**Keywords:** ME/CFS, omics, explainable boosting machine, ensemble learning, feature selection, PRNN

## Abstract

Myalgic encephalomyelitis/chronic fatigue syndrome (ME/CFS) is a debilitating multisystem illness characterised by post-exertional malaise, non-restorative sleep, and cognitive impairment, yet no objective diagnostic biomarkers have been established. Untargeted plasma metabolomics provides a broad view of the biochemical disturbances underlying ME/CFS; however, the high dimensionality of omics datasets and the limited interpretability of conventional classifiers nevertheless hinder translation into clinical practice. This study evaluates three ensemble classifiers—Explainable Boosting Machine (EBM), XGBoost, and LightGBM—for binary ME/CFS classification using plasma metabolomic and lipidomic profiles from 197 participants (106 ME/CFS; 91 healthy controls; 888 features). Feature dimensionality was reduced using a Pareto-Guided Recursive Neural Network (PRNN) pipeline. Model performance was assessed via 50-repeat stratified hold-out validation. EBM achieved the highest accuracy (0.909; 95% CI: 0.868–0.949) and area under the receiver operating characteristic curve (AUC: 0.940; 95% CI: 0.909–0.983), with XGBoost and LightGBM performing comparably. Interpretability analyses revealed that pairwise metabolite interaction terms—particularly proline & indole-3-lactate, tyrosine & *N*-acetylornithine, and maleic acid & arachidic acid—contributed the greatest discriminative signal. An ablation analysis comparing the full interaction-augmented EBM (AUC = 0.940) with a main-effects-only EBM (AUC = 0.882) confirmed that pairwise metabolite co-variation contributes additional discriminative value beyond individual metabolite levels, implicating amino acid catabolism, tryptophan–kynurenine pathway dysregulation, mitochondrial energy impairment, and lipid remodelling as central pathophysiological features. Global and instance-level explanations jointly demonstrated population-level metabolic signatures alongside individual heterogeneity, highlighting the added clinical value of explainable artificial intelligence (XAI) in metabolomics. These findings support EBM-based metabolomic profiling as an internally validated approach for ME/CFS classification, subject to external validation, calibration assessment, and prospective testing.

## 1. Introduction

Myalgic encephalomyelitis/chronic fatigue syndrome (ME/CFS) is a chronic, severely disabling illness characterised by profound fatigue unrelieved by rest, post-exertional malaise (PEM), non-restorative sleep, and cognitive impairment—with functional reductions comparable to or exceeding those seen in multiple sclerosis and end-stage renal disease [1,2]. Diagnosis relies on symptom-based criteria (most notably the 2015 IOM criteria requiring reduced functional capacity, PEM, unrefreshing sleep, and cognitive impairment or orthostatic intolerance [3,4], as no validated objective biomarker exists. This contributes to severe under-recognition: an estimated 91% of US patients remain undiagnosed, and those who are diagnosed typically wait four to five years from symptom onset [2,5,6]. European expert consensus identifies the same pattern across healthcare systems and calls for improved case-finding, standardised diagnostic pathways, and biomarker-driven research [7]. The substantial overlap between ME/CFS and long COVID—a significant proportion of long COVID patients meet ME/CFS criteria and share core pathophysiological features [8,9,10], has intensified interest in objective molecular diagnostic tools.

Untargeted metabolomics has emerged as a leading strategy for identifying ME/CFS biochemical signatures. Naviaux et al. [11] described a broadly hypometabolic plasma profile spanning sphingolipid, phospholipid, purine, and cholesterol pathways, consistent with a dauer-like cellular stress response. Subsequent studies corroborated dysregulation across amino acid catabolism, the TCA cycle, oxidative phosphorylation, and one-carbon metabolism [12]. although sample heterogeneity and methodological variation have complicated cross-study replication. Multi-omic integration of gut metagenomic, plasma metabolomic, and immune profiling data has further expanded the pathophysiological map: Guo et al. [13] and Xiong et al. [14] independently reported reduced microbial butyrate-producing capacity and altered short-chain fatty acid, bile acid, and tryptophan-derived metabolites in large, geographically diverse ME/CFS cohorts, while Xiong et al. [15] recently demonstrated that supervised deep-learning integration of longitudinal multi-omic data can discriminate ME/CFS and identify symptom-specific biomarkers. Metabolomic overlap with long COVID—particularly in sarcosine, serine, aspartate, alanine, and proline pathways [16,17,18], positions ME/CFS within a broader class of post-infectious, energetically compromised states.

Machine learning (ML) complements conventional metabolomic analysis by detecting non-linear and co-varying metabolite signatures inaccessible to univariate methods. Gradient-boosted ensembles such as XGBoost and LightGBM [19,20] have shown consistently strong performance on high-dimensional tabular biomedical data, combining regularisation, efficient handling of missing values, and competitive predictive accuracy; recent metabolomics applications in atherosclerotic cardiovascular disease [21], breast cancer [22], and inborn errors of metabolism screening [23] have established these methods as leading approaches for tabular biomarker discovery. Their principal limitation is opacity: predictions lack feature-level explanations, restricting clinical utility. Explainable Boosting Machines (EBMs)—a glass-box architecture extending generalised additive models with pairwise interaction terms [24,25], address this gap by producing fully auditable, feature-specific contribution scores without sacrificing predictive performance. A complementary challenge is the curse of dimensionality inherent to untargeted metabolomics. The Pareto-Guided Recursive Neural Network (PRNN) approach [17] addresses this through multi-objective Pareto optimisation combined with recursive feature elimination, preserving interaction-prone feature pairs that univariate filters or LASSO penalisation would discard—pairs that are precisely those most relevant to ME/CFS pathophysiology.

Several mechanistic axes inform the interpretation of metabolomic findings in ME/CFS: tryptophan–kynurenine pathway dysregulation linking gut microbial activity, NAD^+^ biosynthesis, and neuroinflammation [26,27,28]. mitochondrial bioenergetic impairment manifesting as reduced oxidative phosphorylation and dysregulated TCA cycle flux [8,18,29], nitrosative and oxidative stress through a self-reinforcing peroxynitrite–NO cycle [30,31], and catecholaminergic dysregulation underpinning orthostatic and exertional symptoms [32]. These frameworks provide the biological context against which classifier findings should be interpreted.

Against this background, the present study pursues three objectives: (i) to evaluate EBM, XGBoost, and LightGBM for binary ME/CFS classification under a rigorous 50-repeat stratified hold-out framework applied to a 197-participant plasma metabolomics cohort; (ii) to characterise the metabolic features driving discrimination at population and individual levels using EBM’s global and local interpretability framework; and (iii) to assess whether pairwise metabolite interaction terms yield biologically informative signal beyond individual-feature analyses. To the best of our knowledge, this is the first integrated PRNN–EBM pipeline applied to ME/CFS metabolomics and the first study to systematically examine pairwise interaction terms as a dedicated source of discriminative signal in this condition.

## 2. Results

### 2.1. Statistical Analysis Result

The cohort comprised 197 participants: 106 with ME/CFS and 91 healthy controls. The two groups were well-matched on demographic variables. The mean age was 47.8 ± 13.7 years in the ME/CFS group and 47.0 ± 14.1 years in controls (*p* = 0.78); mean BMI was 26.1 ± 5.2 and 25.2 ± 4.7 kg/m^2^, respectively (*p* = 0.31); and sex distribution did not differ significantly (31 male, 75 female vs. 22 male, 69 female; *p* = 0.52). Several clinical and medication-related variables differed markedly between groups. Self-reported irritable bowel syndrome (IBS) was substantially more prevalent among ME/CFS participants (35/106, 33.0%) than controls (3/91, 3.3%; *p* < 0.001). Probiotic supplement use was similarly elevated in the ME/CFS group (41/106, 38.7%) relative to controls (10/91, 11.0%; *p* < 0.001). Anti-depressant use was reported by 41 ME/CFS participants (38.7%) versus 12 controls (13.2%; *p* < 0.001), and narcotic pain reliever use was markedly higher in the ME/CFS group (24/106, 22.6%) compared to controls (2/91, 2.2%; *p* < 0.001). Prebiotic supplement use was also more frequent among ME/CFS participants (11/106, 10.4% vs. 2/91, 2.2%; *p* = 0.02). Antibiotic use in the preceding 6–12 weeks did not differ significantly between groups (13/106 vs. 5/91; *p* = 0.14) (Table 1).

In addition, disease severity, assessed using the Multidimensional Fatigue Inventory (MFI), confirmed pronounced symptom burden in the ME/CFS group across all five subscales (all *p* < 0.001). General fatigue scores were 83.3 ± 20.3 in ME/CFS participants versus 22.4 ± 19.5 in controls, and physical fatigue scores were 80.7 ± 20.4 versus 17.3 ± 17.8. Reduced activity was similarly elevated (74.9 ± 22.7 vs. 16.7 ± 19.8). Mental fatigue, which served as a validated proxy for cognitive impairment, was 60.3 ± 23.6 in the ME/CFS group compared to 19.6 ± 21.2 in controls. Reduced motivation scores were 48.4 ± 26.3 versus 20.7 ± 23.5.

### 2.2. Model Performance

Three ensemble classifiers—EBM, XGBoost, and LightGBM—were evaluated under a 50-repeat stratified hold-out scheme; results are summarised in Table 2. Across all five performance metrics, EBM achieved the highest point estimates, followed by XGBoost and then LightGBM. EBM reached an accuracy of 0.909 and an F1-score of 0.913, with sensitivity and specificity both above 0.89, indicating balanced detection of ME/CFS cases and controls. XGBoost and LightGBM performed comparably, with all metrics exceeding 0.86. Importantly, the 95% confidence intervals overlap substantially across the three classifiers; therefore, the small point-estimate differences do not constitute statistical evidence that any single model outperforms the others. Detailed metric values with confidence intervals are reported in Table 2.

To assess the degree of overfitting, mean training AUC was compared with mean test AUC across the 50 iterations. Training AUC values were 0.968 for EBM, 0.978 for XGBoost, and 0.975 for LightGBM, compared with test AUC values of 0.940, 0.931, and 0.929, respectively. The train-to-test gap was modest across all three models (0.028–0.047), consistent with well-controlled regularisation rather than severe overfitting. Notably, EBM exhibited the smallest train-to-test gap despite achieving the highest test AUC, reflecting its additive architecture’s stronger implicit regularisation relative to the full tree-ensemble structures of XGBoost and LightGBM. This pattern—where EBM achieves lower training AUC but higher test AUC than the comparator models—is consistent with superior generalisation capacity and supports its selection as the primary model for interpretability analysis.

### 2.3. ROC and Precision–Recall Analyses

**Figure 1:**
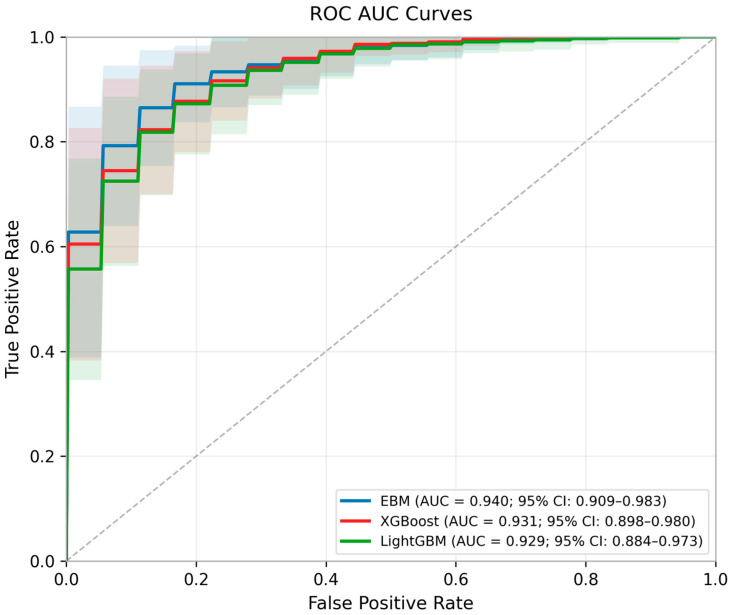
Receiver operating characteristic (ROC) curves for all three classifiers (EBM, XGBoost, and LightGBM).

Figure 1 presents mean ROC curves for all three classifiers across the 50-repeat stratified hold-out validation. For each repetition, ROC curves were computed using only the held-out test partition, interpolated onto a common false-positive-rate grid, and averaged across repetitions. All three models demonstrated strong discriminative ability, with EBM achieving the highest mean AUC of 0.940 (95% CI: 0.909–0.983), followed by XGBoost (AUC = 0.931; 95% CI: 0.898–0.980) and LightGBM (AUC = 0.929; 95% CI: 0.884–0.973). The shaded regions represent empirical 95% confidence bands calculated from the distribution of true-positive-rate values across the 50 repetitions at each false-positive-rate point. AUC confidence intervals were derived from the empirical distribution of per-iteration AUC values.

**Figure 2:**
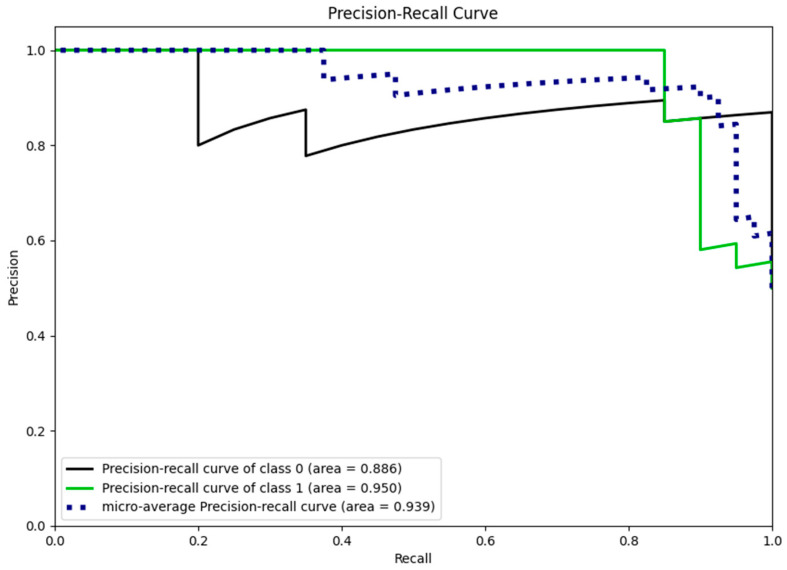
Class-specific precision–recall curves for the EBM model. Class-level presentation was retained to reflect the asymmetric discriminative performance arising from the mildly imbalanced cohort (106 ME/CFS vs. 91 controls). AUPRC: area under the precision–recall curve.

Precision–recall (PR) analysis (Figure 2) yielded a consistent picture. Area under the precision-recall curve (AUPRC) reached 0.886 for the control class and 0.950 for the ME/CFS class. The higher AUPRC for ME/CFS indicates that precision was maintained even at high recall thresholds—a clinically important property given that undetected ME/CFS cases carry greater harm than false-positive referrals.

### 2.4. Feature Selection and Global Feature Importance

PRNN feature selection was applied independently within the training partition at each of the 50 hold-out iterations, prior to model fitting, ensuring no information from the test partition influenced the selection process. PRNN operates exclusively on individual metabolite features: in each iteration, it progressively eliminates redundant features from the original 888-feature space through Pareto-guided recursive elimination, retaining the subset that maximises discriminative performance across multiple evaluation criteria simultaneously. Across the 50 iterations, the number of individual metabolites selected by PRNN ranged from 112 to 134, representing approximately 12.6–15.1% of the original 888-feature space. This variability reflects differences in training-set composition across random splits and is consistent with the expected behaviour of recursive feature elimination methods on high-dimensional omics data.

**Figure 3:**
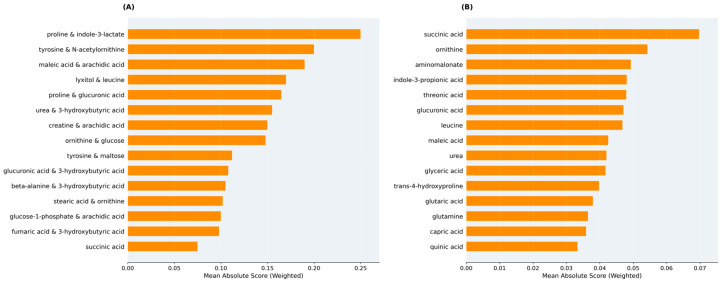
Global term/feature importances derived from the Explainable Boosting Machine (EBM) model. Importance scores are presented as mean absolute weighted contributions to the classification function, averaged across all 50 stratified hold-out iterations. (**A**) Top 15 terms ranked in descending order of their aggregate contribution to ME/CFS classification, including pairwise interaction terms (EBM configured with interactions = ‘auto’; AUC = 0.940, 95% CI: 0.909–0.983). (**B**) Top 15 individual metabolite features from an EBM model trained without interaction terms (interactions = 0; AUC = 0.882, 95% CI: 0.820–0.951). The reduction in AUC when interaction terms are excluded demonstrates that pairwise metabolite co-variation contributes additional discriminative signal beyond individual metabolite levels alone.

It is important to clarify that PRNN selects individual metabolites only; pairwise interaction terms are not selected by PRNN but are generated natively by EBM from the PRNN-selected feature subset during model training. Specifically, EBM was configured with interactions = ‘auto’, which employs the FAST algorithm to evaluate all possible pairwise combinations among the PRNN-selected features and retains only those with statistically meaningful contributions. From a pool of 6216 to 8911 possible pairs per iteration, EBM identified 14 pairwise interaction terms and 1 individual metabolite term as carrying the highest mean absolute weighted contributions across the 50 iterations; these are reported in Figure 3A.

There is therefore no single fixed final feature set across iterations: at each iteration, EBM receives the PRNN-selected individual metabolites for that split and constructs interaction terms autonomously. The global feature importance ranking reported in Figure 3A represents mean absolute weighted contributions averaged across all 50 iterations, which stabilises the reported rankings relative to any single split. The top-ranked feature pair was proline & indole-3-lactate (mean absolute weighted score ≈ 0.25), which substantially outpaced all other terms. Tyrosine & *N*-acetylornithine (≈0.20) and maleic acid & arachidic acid (≈0.19) ranked second and third, respectively. A cluster of features contributed in the 0.15–0.17 range: lyxitol & leucine, proline & glucuronic acid, urea & 3-hydroxybutyric acid, creatine & arachidic acid, and ornithine & glucose. Intermediate contributions (≈0.10–0.12) were observed for tyrosine & maltose, glucuronic acid & 3-hydroxybutyric acid, and beta-alanine & 3-hydroxybutyric acid. Stearic acid & ornithine, glucose-1-phosphate & arachidic acid, fumaric acid & 3-hydroxybutyric acid, and succinic acid exhibited the lowest—yet non-negligible—importance scores. Pairwise interaction terms dominate the top-15 ranking, suggesting that the model’s discriminative power derives principally from metabolite co-variation rather than from individual metabolite levels alone.

To directly assess whether pairwise interaction terms contribute additional discriminative value beyond individual metabolite signals, an EBM model was trained without interaction terms (interactions = 0) under the identical 50-repeat stratified hold-out framework. This main-effects-only model yielded a mean AUC of 0.882 (95% CI: 0.820–0.951), compared with 0.940 (95% CI: 0.909–0.983) for the interaction-augmented model—a reduction of 0.058 AUC units. As shown in Figure 3B, the main-effects-only model identified a distinct feature set dominated by individual metabolites (succinic acid, ornithine, aminomalonate, and indole-3-propionic acid), with the constituent metabolites of the leading interaction pairs, proline, indole-3-lactate, tyrosine, and *N*-acetylornithine, absent from the top-15 rankings. This confirms that the discriminative signal carried by these metabolites emerges from their co-variation rather than from their marginal effects, a signature inaccessible to conventional single-feature or main-effects-only approaches and uniquely captured by EBM’s interaction-aware architecture.

To provide direct supporting evidence at the level of individual metabolites, case-control distributions and univariate association statistics for all 20 metabolites constituting the top-15 EBM-ranked terms are reported in Supplementary Table S1 and Figure S1. Of these 20 metabolites, only five showed nominally significant univariate differences between ME/CFS cases and controls (indole-3-propionic acid, aminomalonate, ornithine, leucine, and succinic acid; all *p* < 0.05), while the majority—including proline and indole-3-lactate, the constituents of the top-ranked interaction term—did not reach univariate significance in isolation (*p* = 0.777 and *p* = 0.237, respectively). This dissociation between weak univariate signal and strong joint discriminative value, detailed in the Supplementary Materials, provides direct empirical support for the conclusion that the model’s discriminative power derives from metabolite co-variation rather than from individual-level effects (Supplementary Table S1 and Figure S1).

### 2.5. Local Explanations

**Figure 4:**
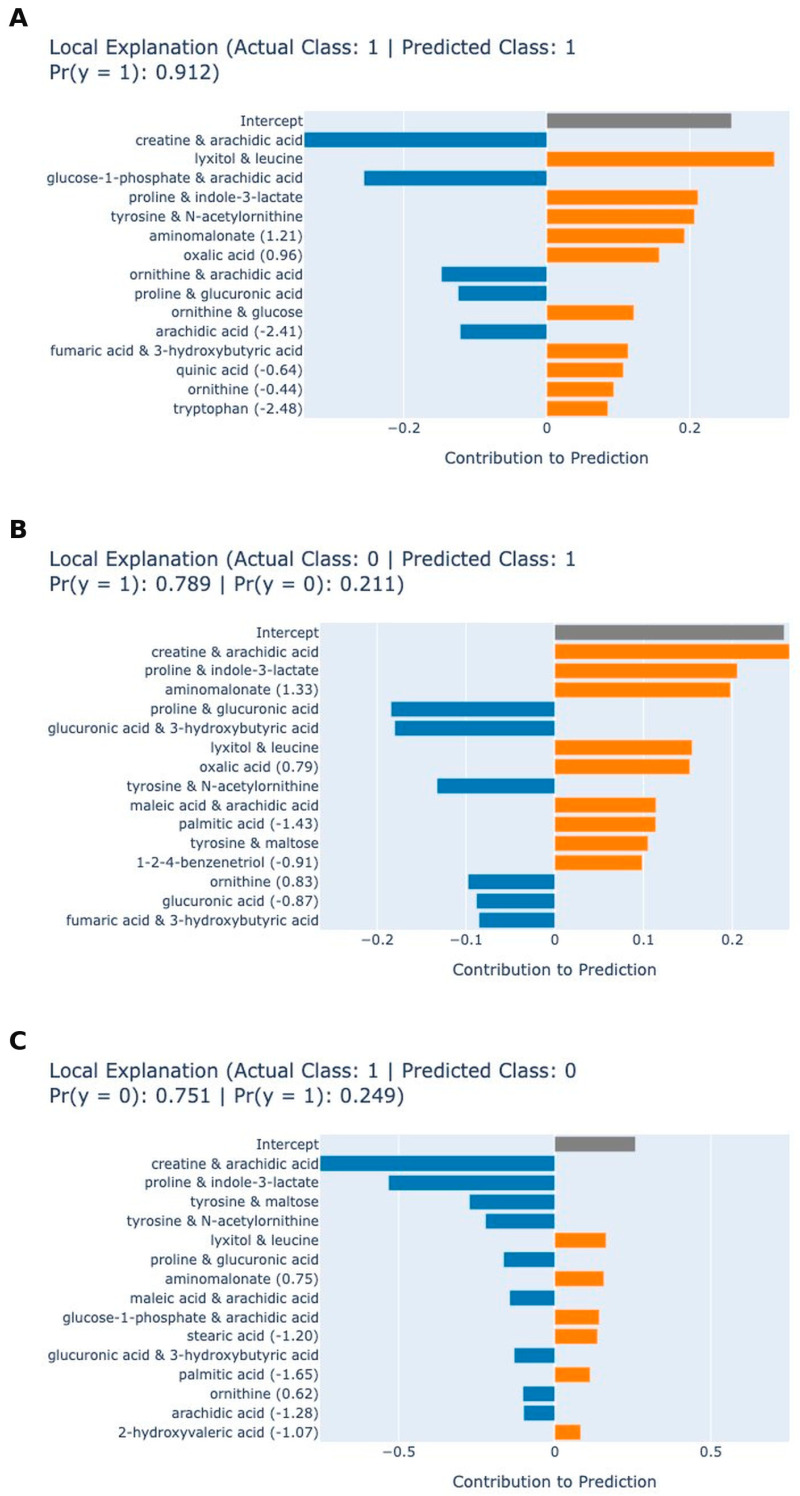
Local explanations generated by the Explainable Boosting Machine (EBM) model for three representative cases. (**A**) True positive (Actual Class: 1|Predicted Class: 1; Pr(y = 1) = 0.912). (**B**) False positive (Actual Class: 0|Predicted Class: 1; Pr(y = 1) = 0.789; Pr(y = 0) = 0.211). (**C**) False negative (Actual Class: 1|Predicted Class: 0; Pr(y = 0) = 0.751; Pr(y = 1) = 0.249). Orange bars indicate features that increased the predicted probability toward ME/CFS; blue bars indicate features that decreased it. The intercept represents the model’s baseline (prior) prediction before individual feature contributions are applied.

Figure 4 presents instance-level EBM explanations for three representative cases, illustrating the directional contribution of individual features to each prediction. In Panel A (true positive; Pr(y = 1) = 0.912), lyxitol & leucine and proline & indole-3-lactate provided the largest positive contributions toward the ME/CFS classification, whereas creatine & arachidic acid and glucose-1-phosphate & arachidic acid most strongly counteracted the ME/CFS prediction. Panel B (false positive; Pr(y = 1) = 0.789) shows that creatine & arachidic acid and proline & indole-3-lactate drove the misclassification toward the positive class, outweighing counter-contributions from proline & glucuronic acid and glucuronic acid & 3-hydroxybutyric acid. Panel C (false negative; Pr(y = 0) = 0.751) illustrates a case in which creatine & arachidic acid, tyrosine & maltose, and tyrosine & *N*-acetylornithine collectively directed the prediction toward the control class, overcoming smaller positive contributions from lyxitol & leucine and other features.

Taken together, these cases demonstrate that global importance rankings capture population-level tendencies, yet each individual prediction is shaped by that patient’s specific metabolic profile. Instance-level explanations therefore add clinical value beyond aggregate rankings, enabling clinicians to trace the reasoning underlying any given classification—a property essential for responsible deployment in diagnostic support contexts.

## 3. Discussion

This study demonstrates that EBM, XGBoost, and LightGBM can discriminate ME/CFS from healthy controls with high accuracy using untargeted plasma metabolomic and lipidomic profiles, following PRNN-based dimensionality reduction. The EBM model achieved the best overall performance (accuracy: 0.909; AUC: 0.940), though the overlapping confidence intervals across all three classifiers indicate that no single model is statistically superior—consistent with the general observation that well-tuned gradient-boosted ensembles tend to converge on similar performance levels in moderately sized biomedical datasets [21,22]. The accuracy and AUC values reported here are comparable to those obtained by recent deep-learning multi-omics frameworks applied to ME/CFS [15], suggesting that well-engineered tabular ensemble methods coupled with interpretability frameworks can achieve competitive performance without the sample-size requirements and interpretability challenges of deep-learning architectures. What distinguishes EBM here is not primarily its predictive accuracy but its interpretability: unlike XGBoost and LightGBM, EBM decomposes predictions into auditable, feature-specific and pairwise-interaction contributions that are clinically communicable without sacrificing performance.

Table 3 represents the present study within the recent literature on machine-learning-based metabolomic classification, comparing it with representative ME/CFS multi-omic and metabolomic-ML studies as well as with metabolomic-ML applications to other complex diseases. Two patterns are notable. First, ME/CFS-specific studies have so far been dominated by descriptive metabolomics [11] and by microbiome- or multi-omic-driven frameworks that achieve high discriminative performance using deep-learning or tree-ensemble models with post-hoc interpretability [13,15]. Second, metabolomic-ML pipelines for other diseases—such as atherosclerotic cardiovascular disease [21] and breast cancer [22]—have already adopted SHAP-based interpretability of black-box ensembles as a de facto standard. The present pipeline differs from both groups along three axes: (i) it employs glass-box, intrinsically interpretable EBM rather than post-hoc-explained black-box ensembles; (ii) it uses PRNN instead of univariate filters or LASSO penalisation, preserving interaction-prone feature pairs; and (iii) it explicitly reports both global and instance-level interaction-term contributions as auditable model outputs. The performance values obtained here (EBM accuracy 0.909, AUC 0.940) are within the range reported by comparable plasma-based metabolomic-ML studies and by deep-learning multi-omic ME/CFS frameworks, while preserving full interpretability of the discriminative signal. Beyond this methodological positioning, the principal advance reported here is biological as well as algorithmic: by exposing pairwise metabolite interactions as auditable, glass-box contributions, the pipeline reframes ME/CFS classification around metabolite co-variation and links the leading interaction terms to specific mechanistic axes (mitochondrial bioenergetics, the tryptophan–kynurenine pathway, and the urea–NO axis), thereby converting a predictive model into a source of mechanistically interpretable, clinically testable hypotheses.

Before interpreting these findings biologically, we emphasise that the EBM pairwise terms quantify statistical co-variation between metabolites within the fitted classifier and do not, in themselves, denote direct biochemical or causal interactions; the mechanistic correspondences proposed in the following paragraphs are therefore offered as biologically motivated, hypothesis-generating interpretations rather than demonstrated molecular relationships. The dominance of pairwise interaction terms in the global feature importance ranking was further validated by an ablation analysis. An EBM model trained without interaction terms under the identical pipeline yielded a mean AUC of 0.882 (95% CI: 0.820–0.951), compared with 0.940 (95% CI: 0.909–0.983) for the interaction-augmented model—a reduction of 0.058 AUC units. Critically, the constituent metabolites of the leading interaction pairs (proline, indole-3-lactate, tyrosine, *N*-acetylornithine) were absent from the top-15 rankings of the main-effects-only model, which instead identified succinic acid, ornithine, and aminomalonate as the dominant individual features. This dissociation confirms that the discriminative signal carried by the leading metabolite pairs emerges exclusively from their co-variation, a signature that is invisible to conventional single-feature or main-effects-only approaches and uniquely captured by EBM’s interaction-aware architecture.

The predominance of pairwise interaction terms in the global feature importance ranking is biologically informative. The top-ranked pair, proline & indole-3-lactate, implicates two interconnected metabolic axes of considerable relevance to ME/CFS pathophysiology. Proline metabolism is tightly linked to mitochondrial redox balance: proline dehydrogenase/oxidase (PRODH/POX) localises to the inner mitochondrial membrane and transfers electrons directly to ubiquinone, thereby coupling proline catabolism to the electron transport chain and ATP synthesis while simultaneously modulating reactive oxygen species (ROS) generation [33,34,35]. Importantly, PRODH/POX activity is co-regulated by succinate, which exerts uncompetitive inhibition on PRODH-mediated respiration [35]—a cross-talk that may be particularly informative given that succinic acid appears as an independent feature in the EBM importance ranking. Dysregulation of proline catabolism is consistent with the mitochondrial dysfunction hypothesis of ME/CFS, which posits impaired oxidative phosphorylation as a central energy-limiting mechanism [8,11,18], and with recent genome-scale metabolic modelling that identified arginine–proline pathway downregulation as a shared metabolic signature of ME/CFS and long COVID [36]. Indole-3-lactate is a tryptophan-derived metabolite produced in part through intestinal microbial activity, reflecting the state of the kynurenine pathway and gut–brain axis signalling [26]. The IDO1/IDO2-mediated tryptophan catabolism cascade—which generates kynurenine and downstream neuroactive metabolites, including quinolinic acid—has been proposed as a mechanism linking microbiome dysbiosis to neuroinflammation, NAD^+^ depletion, and cognitive impairment in ME/CFS [27,28]. The co-variation of proline and indole-3-lactate as the strongest discriminating pair implicates both mitochondrial bioenergetics and gut–brain axis signalling, consistent with the multi-omic picture emerging from contemporary ME/CFS research.

The second-ranked interaction, tyrosine & *N*-acetylornithine, connects catecholamine biosynthesis with urea cycle metabolism. Tyrosine is the immediate precursor for dopamine, norepinephrine, and epinephrine; perturbations in tyrosine availability or enzymatic conversion can impair autonomic function and central neurotransmitter balance—both of which are clinically compromised in ME/CFS, where orthostatic intolerance and postural tachycardia syndrome are frequent comorbidities [32]. *N*-acetylornithine is an intermediate in the ornithine–arginine–urea axis, which is closely coupled to nitric oxide (NO) synthesis via arginine-dependent NO synthase. Chronic elevation of NO and peroxynitrite and consequent hypernitrosylation of cysteine-containing enzymes within the mitochondrial electron transport chain and antioxidant systems have been proposed as a self-perpetuating mechanism linking infection, autoimmunity, and mitochondrial dysfunction in ME/CFS [30,31]. The reported reduction in circulating nitrite/nitrate species and concomitant elevation of endothelial dysfunction markers (ET-1, VCAM-1) in ME/CFS patients compared with healthy controls [37] further supports the proposed coupling between arginine–ornithine–NO dysregulation and the symptomatology of the disease. The joint dysregulation of tyrosine and *N*-acetylornithine as a classification signal reinforces the view that neuroendocrine perturbations in ME/CFS are tightly intertwined with broader metabolic dysfunction of the urea–NO axis.

The recurrence of 3-hydroxybutyric acid across multiple high-ranking interaction terms (urea & 3-hydroxybutyric acid; glucuronic acid & 3-hydroxybutyric acid; beta-alanine & 3-hydroxybutyric acid; fumaric acid & 3-hydroxybutyric acid) is consistent with impaired mitochondrial fatty acid β-oxidation and deficient glucose oxidation. 3-Hydroxybutyrate is a ketone body that accumulates when mitochondrial acetyl-CoA generation from glucose is insufficient to meet cellular energy demands; its prominent role across feature pairs suggests a systemic shift toward alternative energy substrates in ME/CFS patients, closely aligned with the hypometabolic state described by Naviaux et al. [11] and with the post-exertional downregulation of TCA cycle–related extracellular vesicle proteins reported by Glass et al. [18]. These biochemical shifts coincide physiologically with reduced systemic oxygen extraction and attenuated oxidative phosphorylation capacity during exertion [8] and have motivated the exploration of blood lactate and ketone body monitoring as candidate indicators of post-exertional malaise [29]. Similarly, the appearance of creatine & arachidic acid as a highly ranked pair reflects the dual perturbation of phosphocreatine-based ATP buffering and long-chain saturated fatty acid metabolism—pathways intimately connected to cellular energy homeostasis and membrane integrity and consistent with the broader one-carbon and creatine pathway perturbations documented in cerebrospinal fluid metabolomics of ME/CFS [12].

The appearance of tyrosine & maltose and ornithine & glucose among high-importance pairs is notable in the context of microbiome–host metabolic interactions. Guo et al. [13] and Xiong et al. [14] identified reduced butyrate-producing microbial capacity and altered carbohydrate fermentation products in ME/CFS patients from the same and related cohorts and ascribed a portion of the observed metabolomic variance to microbial metabolism. The detection of carbohydrate- and amino-acid-centred interaction pairs within our model is therefore consistent with the hypothesis that gut microbial metabolism contributes substantively to the plasma metabolic fingerprint of ME/CFS [9] and provides a mechanistic rationale for multi-omic integration in future work.

The PRNN feature selection framework contributed meaningfully to model performance by reducing the 888-feature space to a compact, biologically informative subset without imposing prior biochemical class constraints. The Pareto-guided multi-objective optimisation at the core of PRNN avoids the instability of single-criterion ranking methods and is particularly well-suited to omics data, where features are often collinear and individual importances are unstable across resampling folds [17]. By retaining interaction-prone feature pairs rather than discarding them as collinear noise, PRNN likely facilitated EBM’s capacity to capture metabolite co-variation as a primary classification signal.

The local explanation analyses (Figure 4) revealed substantial individual heterogeneity. In the false-negative case, the combined negative contributions of creatine & arachidic acid, tyrosine & maltose, and tyrosine & *N*-acetylornithine collectively overwhelmed positive contributions from other pairs, leading to misclassification. This underscores a clinically important principle: ME/CFS is likely not a biochemically homogeneous condition; patients may present with distinct metabolic subtypes in which canonical discriminating features deviate from population norms [10,15]. Instance-level explanations are therefore not merely technical artefacts of interpretability but constitute genuine clinical information that could guide personalised diagnostic approaches.

The integration of global and instance-level interpretability analyses exposed individual heterogeneity in ME/CFS metabolic profiles and demonstrated that population-level rankings are insufficient to fully explain individual predictions. This carries direct translational implications: clinician-facing diagnostic tools must communicate not only which metabolites are collectively abnormal in ME/CFS but also why a particular patient was classified as they were. Analogous XAI-driven metabolomic frameworks in atherosclerotic cardiovascular disease [21] and breast cancer [22] have demonstrated that pairing ensemble classifiers with SHAP or native interpretability modules materially improves clinician trust and biomarker prioritisation. EBM satisfies this requirement in a manner that black-box ensemble methods do not, and our results suggest that the interpretability advantage does not come at the cost of predictive accuracy for ME/CFS classification.

This study has several limitations that deserve mention. The cohort comprised 197 participants—106 ME/CFS cases and 91 healthy controls—drawn from a single multi-site collection whose composition overlaps with that used in prior microbiome studies of ME/CFS [13]; independent external validation in geographically and clinically diverse cohorts, including long COVID populations, is required before clinical translation can be contemplated. Second, the retrospective cross-sectional design precludes causal inference; the metabolic signatures identified are discriminatory associations, not confirmed mechanistic biomarkers [37]. Third, EBM’s interaction terms, while interpretable, reflect statistical co-variation rather than causal metabolic relationships and should be regarded as hypothesis-generating. Fourth, the absence of a replication cohort means that confidence intervals reflect internal variability only. Fifth, although the IOM diagnostic criteria are widely accepted, residual diagnostic heterogeneity is inevitable in symptom-defined cohorts and may dilute or exaggerate specific metabolic signals [5,7]. Future work should focus on external validation in independent cohorts, longitudinal metabolomic profiling to assess biomarker stability, and multimodal integration with genomic, microbiome, immune, and clinical phenotyping data to contextualise the metabolic signatures identified here [15]. Sixth, although cases and controls were frequency-matched by sex, age, race/ethnicity, and geographic/clinical site at enrolment, and participants with significant confounding conditions or immunomodulatory drug use were excluded at enrolment, non-metabolomic variables including body mass index, detailed medication use, collection site, season of collection, and comorbidities were not incorporated as explicit inputs into the machine learning models. This decision was consistent with the primary objective of the study to develop and evaluate a metabolite- and lipid-driven explainable classification pipeline and with the established practice of restricting model inputs to the omics feature space in biomarker discovery analyses. Nevertheless, residual confounding from site-level technical variation, seasonal metabolomic fluctuations, medication effects on the plasma metabolome, and other demographic and clinical variables cannot be fully excluded. Reported classification performance and feature importance rankings should therefore be interpreted as cohort-specific, exclusion-filtered associations rather than covariate-adjusted effect estimates; quantifying the influence of these variables will require prospectively pooled cohorts with complete covariate information and explicit adjustment in both preprocessing and modelling stages.

## 4. Materials and Methods

### 4.1. Study Design and Dataset

We conducted a retrospective analysis of an existing, de-identified omics dataset. Plasma metabolomic and lipidomic profiles were available for 197 participants: 106 meeting ME/CFS diagnostic criteria and 91 healthy controls, drawn from a multi-site, geographically diverse North American collection previously described in the context of gut microbiome investigations of ME/CFS. Diagnoses were established using the 2015 Institute of Medicine (IOM) criteria, requiring a significant reduction in functional capacity, post-exertional malaise, non-restorative sleep, and either cognitive impairment or orthostatic intolerance [4]. Exclusion criteria were applied at enrolment to limit confounding: participants with chronic infections, rheumatic or inflammatory diseases, neurological disorders, active psychiatric conditions, or current immunomodulatory drug use were not included. Blood was drawn after overnight fasting into BD Vacutainer™ Cell Preparation Tubes (Franklin Lakes, NJ, USA) with EDTA anticoagulant, immediately centrifuged at 4 °C, aliquoted, and stored at −80 °C until analysis. Healthy controls were matched to ME/CFS cases on sex, age, geographic and clinical region, and race/ethnicity. Potential confounding was thus addressed primarily at the design stage through case–control matching on sex, age, geographic and clinical region, and race/ethnicity and through the enrolment exclusions listed above (chronic infection; inflammatory, neurological, and active psychiatric disease; and current immunomodulatory drug use) [13,38].

### 4.2. Metabolomic and Lipidomic Profiling and Feature Space

The dataset contains 888 metabolic and lipid features spanning amino acids, lipids, organic acids, nucleotides, carbohydrates, cofactors, vitamins, and xenobiotics. Quantification was performed on plasma samples using untargeted global metabolomics platforms covering both hydrophilic and lipophilic fractions, consistent with established practice in plasma metabolomics profiling of ME/CFS [11,13,38]. The full feature space was retained in its entirety rather than pre-filtered by biochemical class, enabling the models to detect both individual metabolite signals and co-varying signatures without imposing prior biological assumptions that could introduce selection bias.

### 4.3. Data Preprocessing and Normalisation

An 888-feature plasma metabolomic and lipidomic dataset was generated and quality-controlled using four analytical platforms: (1) GC-TOF MS for primary metabolites processed using the BinBase pipeline; (2) HILIC-QTOF MS for biogenic amines; (3) CSH-QTOF MS for complex lipids; and (4) targeted LC-MS/MS for oxylipins. A total of 821 known metabolites were identified; some complex lipids were detected in both positive and negative ion modes, yielding a total of 888 analytes. Signal intensity normalisation across all four platforms was performed using SERRF (Systematic Error Correction Using Random Forest), a machine learning-based method that corrects batch-to-batch drift, plate effects, and run-order deviations by utilising quality control (QC) samples injected at regular intervals throughout each analytical run. For the GC-TOF MS panel, BinBase also applies FAME-based retention index alignment to ensure chromatographic consistency between runs, regardless of batch. All LC-MS/MS panels incorporated various internal standards to support quantitative accuracy. The remaining technical error was assessed using the coefficient of variation (CV) across known metabolites following SERRF normalisation. Metabolomic profiling was performed independently of the case-control status to eliminate analyst-revealed bias. Zero values reflecting measurements below the detection limit were replaced with 50% of the smallest observed value for that feature (half minimum assignment), a standard approach for GC-MS and LC-MS metabolomic data. Features with a high rate of missing data were excluded during the upstream BinBase process before dataset storage [38]. Therefore, the stored 888-feature dataset represents the quality-controlled, complete output of this pipeline. After dataset acquisition, a log_2_ transformation was applied to reduce rightward skewness in abundance distributions and to balance variance across the feature range. Next, features were subjected to z-score normalisation (mean = 0; standard deviation = 1) to ensure that differences in the measurement scale did not disproportionately affect model learning. Both transformations were calculated only in the training section and applied to the corresponding test section in each of the 50 retention iterations, thus preventing information leakage.

### 4.4. Feature Selection: Pareto-Guided Recursive Neural Network (PRNN)

Given the high dimensionality of the omics dataset (888 features; 197 samples), feature selection was performed using the PRNN embedded approach [17]. PRNN integrates a Pareto-guided Neural Network embedded method (PNN) with a Recursive Feature Elimination (RFE) framework in a single pipeline, progressively discarding redundant features while retaining those with the strongest discriminative signal.

In the first stage, a feedforward neural network is trained on the full feature set, and initial importance scores are derived from the learned weights. These scores are refined through Pareto optimisation, which evaluates features across multiple criteria simultaneously rather than collapsing performance to a single scalar. A feature is Pareto-optimal when no other feature dominates it across all evaluation criteria simultaneously—a condition that yields more stable, multi-criteria-informed rankings than single-objective filtering. In the second stage, the PNN-derived Pareto scores are fed into an RFE loop: at each iteration, the lowest-ranked features are eliminated, and the remaining feature subset is evaluated using a Support Vector Machine (SVM) classifier with 5-fold cross-validation as an internal wrapper criterion; the subset yielding the highest cross-validation accuracy is retained. This recursive process continues until the feature set is reduced to ten or fewer features, at which point the best-performing subset is returned. The SVM serves exclusively as an internal evaluation mechanism within the PRNN pipeline and is not used for final classification; the selected feature subset is subsequently passed to EBM, XGBoost, and LightGBM. PRNN hyperparameters were set as follows: hidden layers = 2 (64 and 32 neurons), activation = ReLU, optimiser = Adam, epochs = 100. The Pareto selection ratio *p* was initialised at 0.5 and adjusted dynamically at each recursive step. Unlike standard RFE—which ranks features by shallow model weights or permutation importance—PRNN applies Pareto-refined neural network scores at every elimination step, thereby accounting for both individual contributions and inter-feature dependencies. PRNN feature selection was applied exclusively within the training partition at each of the 50 hold-out iterations, prior to model fitting. The selected feature subset was then used to transform both the training and test partitions for that iteration. This procedure ensures that no information from the test set influenced the feature selection process, thereby preventing optimistic bias in performance estimates, consistent with best practice in computational omics.

### 4.5. Model Development: Ensemble Learning Classifiers

Three gradient-based ensemble algorithms were employed for ME/CFS classification: EBM, XGBoost [19], and LightGBM [20]. EBM, implemented via the InterpretML framework [25], extends generalised additive models by incorporating pairwise interaction terms and assigns fully interpretable scores to each feature and feature pair [24], making it well-suited to clinical applications where transparency matters. To directly evaluate the contribution of pairwise interaction terms to classification performance, an additional EBM model was trained without interaction terms (interactions = 0) under the identical PRNN–EBM pipeline and 50-repeat stratified hold-out framework. All preprocessing, feature selection, and evaluation procedures were held constant, with the sole difference being the exclusion of interaction terms from the EBM architecture. This main-effects-only model served as a within-framework ablation control, allowing direct quantification of the discriminative value added by pairwise metabolite co-variation beyond individual metabolite signals. XGBoost employs a regularised gradient boosting framework with second-order optimisation and L1/L2 regularisation, handling sparse data efficiently while limiting overfitting. LightGBM grows trees leaf-wise rather than level-wise and uses histogram-based binning, enabling markedly faster training on large feature sets without meaningful loss in accuracy. Together, these three algorithms represent current best practice for tabular biomedical classification and have been widely used in metabolomics-driven biomarker discovery [21,22,23].

Each model was tuned via grid search with 5-fold cross-validation, applied independently within the training partition at each of the 50 hold-out iterations. For XGBoost and LightGBM, the hyperparameter search space covered the number of estimators (100, 200, 500), maximum tree depth (3, 5, 7), learning rate (0.01, 0.05, 0.10), subsampling ratio (0.6, 0.8, 1.0), and regularisation coefficients (L1: 0, 0.1, 1.0; L2: 0, 0.1, 1.0). For EBM, the search space covered the number of boosting rounds (100, 200, 500) and learning rate (0.01, 0.05, 0.10). At each iteration, the optimal configuration was identified exclusively from the training partition and applied to the corresponding held-out test partition, ensuring complete separation between hyperparameter selection and model evaluation.

### 4.6. Model Evaluation: Repeated Hold-Out Validation

Model performance was assessed using a 50-iteration repeated stratified hold-out procedure. At each iteration, the dataset was randomly partitioned 80%/20% into training and test subsets, stratified by class label to preserve the ME/CFS-to-control ratio in both partitions. Performance metrics computed at each iteration included accuracy, F1-score, sensitivity, specificity, and AUC-ROC; final estimates are reported as means with 95% confidence intervals derived from the empirical distribution over 50 repetitions. Fifty iterations were chosen to stabilise estimates and reduce sensitivity to any individual random split—a precaution of particular importance given the modest cohort size.

### 4.7. Discriminative Ability: ROC and Precision–Recall Analyses

For all three classifiers (EBM, XGBoost, and LightGBM), ROC curves were generated by computing the out-of-sample ROC curve separately within each held-out test partition. Each curve was interpolated onto a common false-positive-rate grid, and the mean true-positive-rate curve was obtained by averaging across the 50 stratified hold-out repetitions. Empirical 95% confidence bands were calculated at each false-positive-rate point using the 2.5th and 97.5th percentiles of the corresponding true-positive-rate values across repetitions. AUC confidence intervals were calculated from the empirical distribution of the 50 per-repetition AUC values. Training-set predictions were not used for ROC curve construction. For the EBM model, precision–recall (PR) curves were additionally generated per class to characterise class-specific performance separately for the ME/CFS and control classes. Class-specific PR reporting was retained because the cohort is mildly but clinically imbalanced (106 ME/CFS vs. 91 healthy controls), and the model’s discriminative behaviour may differ between the two classes. In this setting, class-level PR curves provide complementary information beyond ROC analysis, particularly for evaluating performance in the ME/CFS-positive class, where missing a genuine case is more clinically consequential than an incorrect positive referral. Joint reporting of ROC and PR metrics is increasingly recommended for imbalanced or asymmetric medical classification settings [21].

### 4.8. Interpretability Analysis: Global and Local Feature Importance

Since the clinical objective is to identify metabolic signatures with potential biomarker relevance, interpretability analyses were conducted at both population and individual levels using EBM’s native explanation framework. At the global level, term importance scores were extracted for all features and pairwise interaction terms, quantified as mean absolute weighted contributions across the full dataset. These scores provide a biologically grounded ranking of the most discriminative metabolites and metabolite combinations. At the individual level, instance-specific explanations were generated for three representative cases: a true positive (ME/CFS patient correctly classified), a false positive (control misclassified as ME/CFS), and a false negative (ME/CFS patient misclassified as control). For each case, the directional contribution of every feature to the predicted probability was decomposed and visualised, enabling inspection of how that individual’s metabolic profile shaped the model output. This combination of global and local explanations follows current best practice in XAI for clinical machine learning [22]. Term importance scores presented represent mean absolute weighted EBM contributions averaged across all 50 stratified hold-out iterations, rather than values derived from a single split. This averaging procedure stabilises the rankings by suppressing terms with high cross-iteration variance while elevating terms that contribute consistently across partitions, providing a robust estimate of global feature importance.

### 4.9. Statistical Analysis

Differences in cohort characteristics between ME/CFS cases and healthy controls were assessed using the Mann–Whitney U test for continuous variables (age, BMI, and MFI subscale scores) and Fisher’s exact test for categorical variables (sex, comorbidities, and medication and supplement use). A two-sided *p*-value of <0.05 was considered statistically significant. Results are reported as mean ± standard deviation for continuous variables and as counts with percentages for categorical variables.

### 4.10. Software and Computational Environment

All analyses were implemented in Python 3.10. EBM was executed via InterpretML (v0.5.1); XGBoost via xgboost (v1.7.6); LightGBM via lightgbm (v4.0.0). Preprocessing, feature engineering, and evaluation pipelines used scikit-learn (v1.3.0), pandas (v2.0.3), and NumPy (v1.24.4). ROC curves, PR curves, and feature importance plots were generated with matplotlib (v3.7.2) and seaborn (v0.12.2). All experiments were executed on a machine with 32 GB RAM. The analysis code used in this study is publicly available at https://github.com/drhilal/ME-CFS-study (accessed on 2 June 2026).

## 5. Conclusions

Interpretable classification of ME/CFS from healthy controls is achievable using untargeted plasma metabolomics combined with PRNN-based feature selection and EBM. All three classifiers—EBM, XGBoost, and LightGBM—exceeded 0.88 accuracy and 0.92 AUC under rigorous 50-repeat stratified hold-out validation, with EBM attaining the best performance (accuracy: 0.909; AUC: 0.940). The overlapping confidence intervals across classifiers confirm that EBM’s selection as the primary model rests on its interpretability rather than a statistically significant accuracy advantage.

EBM’s glass-box architecture revealed that discriminative power arose predominantly from pairwise metabolite interaction terms rather than individual metabolite levels, implicating metabolic co-variation across amino acid catabolism, mitochondrial energy metabolism, tryptophan–kynurenine pathway activity, and lipid remodelling as central biochemical features of ME/CFS. An ablation analysis directly confirmed the added value of interaction terms: a main-effects-only EBM trained under the identical pipeline yielded a mean AUC of 0.882—a reduction of 0.058 relative to the interaction-augmented model—with some metabolites of the leading interaction pairs absent from the top-15 individual-feature rankings. This dissociation confirms that the discriminative signal identified here reflects genuine metabolite co-variation, not a reframing of individual metabolite effects, and would be inaccessible to conventional single-feature approaches.

The integration of global and instance-level interpretability analyses exposed individual heterogeneity in ME/CFS metabolic profiles and demonstrated that population-level rankings alone are insufficient to explain individual predictions. Clinician-facing diagnostic tools must communicate not only which metabolites are collectively abnormal but also why a particular patient was classified as they were—a requirement that EBM satisfies where black-box ensemble methods fall short.

Future work should focus on independent external validation in geographically and clinically diverse cohorts, longitudinal profiling to assess the stability of identified metabolic signatures, and multimodal integration with genomic, microbiome, and clinical phenotyping data to distinguish causally contributory from merely correlative metabolic signals. The present findings support the continued development of interpretable, metabolomics-driven decision-support tools for ME/CFS and make a case for wider adoption of XAI methods in clinical omics research.

## Figures and Tables

**Table 1:** Descriptive Statistics.

Variable	ME/CFS	Control	*p*-Value
Demographics			
Age (years)	47.8 ± 13.7	47.0 ± 14.1	0.78
BMI (kg/m^2^)	26.1 ± 5.2	25.2 ± 4.7	0.31
Sex			
Male	31	22	0.52
Female	75	69	
Comorbidities			
Self-reported IBS			
Yes	35	3	<0.001
No	71	88	
Medication & supplement use			
Antibiotic use, 6–12 weeks			
Yes	13	5	0.14
No	93	86	
Prebiotic supplement use			
Yes	11	2	0.02
No	95	89	
Probiotic supplement use			
Yes	41	10	<0.001
No	65	81	
Anti-depressant use			
Yes	41	12	<0.001
No	65	79	
Narcotic pain reliever use			
Yes	24	2	<0.001
No	82	88	
Disease characteristics (ME/CFS only)			
Duration > 3 years			
Yes	86	—	—
No	8	—	

Note: Mann-Whitney U test was used for continuous variables; Fisher’s exact test was used for categorical variables. One participant had a missing response for narcotic pain reliever use; twelve participants had a missing response for disease duration.

**Table 2:** Classification performance of EBM, XGBoost, and LightGBM models evaluated on the test set. Results are reported as mean values with 95% confidence intervals (50-repeat stratified hold-out). AUC: area under the receiver operating characteristic curve.

Metric	EBM	XGBoost	LightGBM
Accuracy	0.909 (0.868–0.949)	0.898 (0.856–0.941)	0.883 (0.838–0.928)
F1-Score	0.913 (0.874–0.953)	0.900 (0.862–0.944)	0.889 (0.845–0.933)
Sensitivity	0.896 (0.822–0.947)	0.877 (0.799–0.933)	0.868 (0.788–0.926)
Specificity	0.923 (0.848–0.969)	0.920 (0.837–0.956)	0.901 (0.821–0.954)
AUC	0.940 (0.909–0.983)	0.931 (0.898–0.980)	0.929 (0.884–0.973)

**Table 3:** Comparative summary of recent machine-learning studies on ME/CFS and metabolomic classification of related complex diseases. The present study is reported in the final row. ME/CFS = myalgic encephalomyelitis/chronic fatigue syndrome; CVD = atherosclerotic cardiovascular disease; SHAP = SHapley Additive exPlanations; EBM = Explainable Boosting Machine; PRNN = Pareto-Guided Recursive Neural Network; XAI = explainable artificial intelligence.

Study	Disease/Cohort	ML Model (s)	Feature Selection/Interpretability	Reported Performance
[11]	ME/CFS plasma metabolomics	Multivariate statistics; descriptive metabolomics	Pathway-level analysis; no model-level XAI	Identified hypometabolic ME/CFS signature
[13]	ME/CFS multi-omic (microbiome + metabolome)	Different state-of-the-art ML models	Variable-importance; pathway enrichment; no pairwise interactions	High discriminative performance reported on the relevant task
[15]	ME/CFS longitudinal multi-omic	Supervised deep-learning multi-omic integration	Embedded feature attribution; symptom-specific biomarker discovery	Strong discriminative performance; symptom-axis stratification
[21]	Atherosclerotic CVD plasma metabolomics	Gradient-boosted ensembles (XGBoost, etc.) with SHAP	Domain-knowledge integration; post-hoc SHAP attribution	Competitive AUC reported with explainable feature attribution
[22]	Breast cancer serum metabolomics	LightGBM, AdaBoost, and Random Forest with multi-objective feature selection and SHAP	Multi-objective FS; post-hoc SHAP attribution	High performance metrics with model-agnostic interpretability
Present study	ME/CFS plasma metabolomics	EBM (glass-box), XGBoost, LightGBM	PRNN (Pareto-guided multi-objective FS); intrinsic EBM XAI with global + instance-level explanations and pairwise interaction terms	EBM accuracy 0.909, AUC 0.940

## Data Availability

The raw data supporting the conclusions of this article will be made available by the authors on request.

## Supplementary Materials

The following supporting information can be downloaded at: https://www.mdpi.com/article/10.3390/ijms27135920/s1.
