# Supplementary material for: Metabolomic Classification of Myalgic Encephalomyelitis/Chronic Fatigue Syndrome via Explainable Ensemble Learning and Pareto-Guided Feature Selection
Source: Int J Mol Sci. 2026 Jun 30;27(13):5920. doi: 10.3390/ijms27135920 (PMC13362375; doi:10.3390/ijms27135920)
Supplement: Supplementary file 1 [file ijms-27-05920-s001.zip › ijms-4339894-supplementary.pdf]

Supplementary Materials

Table S1. Case-Control Distributions and Univariate Association Statistics for Top-Ranked Metabolites

Descriptive statistics, effect direction, and univariate association results for the individual metabolites comprising the top-15 ranked terms identified by the Explainable Boosting Machine (EBM) (Figure 3A and 3B), including both individual feature terms and the constituent metabolites of the leading pairwise interaction terms. Values represent raw (pre-transformation) peak intensities, reported as median (interquartile range, IQR) for ME/CFS cases (n = 106) and healthy controls (n = 91), consistent with the non-parametric Mann-Whitney U test used for between-group comparison. Percentage change is calculated relative to the control median.

Metabolite	ME/CFS Median (IQR)	Control Median (IQR)	% Change	Direction	P value*
indole-3-propionic acid	1365.5 (939.5–2012.0)	1806.0 (1139.0–2684.5)	-24.4%	Down in ME/CFS	**0.008**
aminomalonate	3395.0 (2163.5–4603.3)	2672.0 (1718.5–3777.5)	+27.1%	Up in ME/CFS	**0.012**
ornithine	15855.5 (11477.8–27903.5)	20324.0 (14838.0–28030.0)	-22.0%	Down in ME/CFS	**0.018**
leucine	65675.5 (54446.5–83634.5)	73827.0 (60227.0–92418.5)	-11.0%	Down in ME/CFS	**0.018**
succinic acid	2376.0 (1787.8–2927.5)	2015.0 (1501.0–2529.5)	+17.9%	Up in ME/CFS	**0.025**
glucuronic acid	1055.0 (843.3–1437.3)	965.0 (786.0–1210.0)	+9.3%		0.081
maleic acid	843.5 (609.3–1048.0)	750.0 (535.5–991.0)	+12.5%		0.117
tyrosine	41636.0 (33410.3–48673.3)	44765.0 (36319.5–51916.0)	-7.0%		0.117
urea	766044.0 (581075.8–937542.0)	801778.0 (665284.5–976007.5)	-4.5%		0.171
stearic acid	355174.5 (284336.5–416784.0)	355014.0 (300541.0–449497.5)	+0.0%		0.202
N-acetylornithine	2208.0 (1776.0–2675.3)	2458.0 (1773.0–2784.5)	-10.2%		0.210
indole-3-lactate	1812.5 (1527.5–2220.3)	1992.0 (1551.5–2407.0)	-9.0%		0.237
3-hydroxybutyric acid	22068.0 (14081.3–51071.8)	19656.0 (12146.0–38150.5)	+12.3%		0.320
lyxitol	2962.0 (2432.5–3580.8)	3104.0 (2550.0–3661.0)	-4.6%		0.336
beta-alanine	1174.5 (836.5–1490.0)	1236.0 (872.5–1568.0)	-5.0%		0.402
maltose	1094.5 (826.5–1651.3)	1060.0 (858.0–1452.5)	+3.3%		0.621
fumaric acid	9006.5 (7220.0–11074.8)	9409.0 (7372.5–11355.5)	-4.3%		0.640
proline	7945.0 (5024.8–12113.8)	8144.0 (5239.0–12928.5)	-2.4%		0.777
glucose-1-phosphate	2624.0 (2125.8–3450.0)	2707.0 (2120.0–3383.5)	-3.1%		0.876
creatine	6761.5 (4192.0–10070.8)	6401.0 (4469.5–9608.5)	+5.6%		0.917
arachidic acid	4451.0 (3679.3–5703.8)	4800.0 (3277.5–5780.0)	-7.3%		0.922
glucose	370219.0 (308764.0–438161.8)	380837.0 (299942.0–446084.0)	-2.8%		0.989

**P values were derived from two-sided Mann-Whitney U tests comparing raw metabolite abundance between ME/CFS cases and healthy controls; values in bold red indicate $p < 0.05$.*

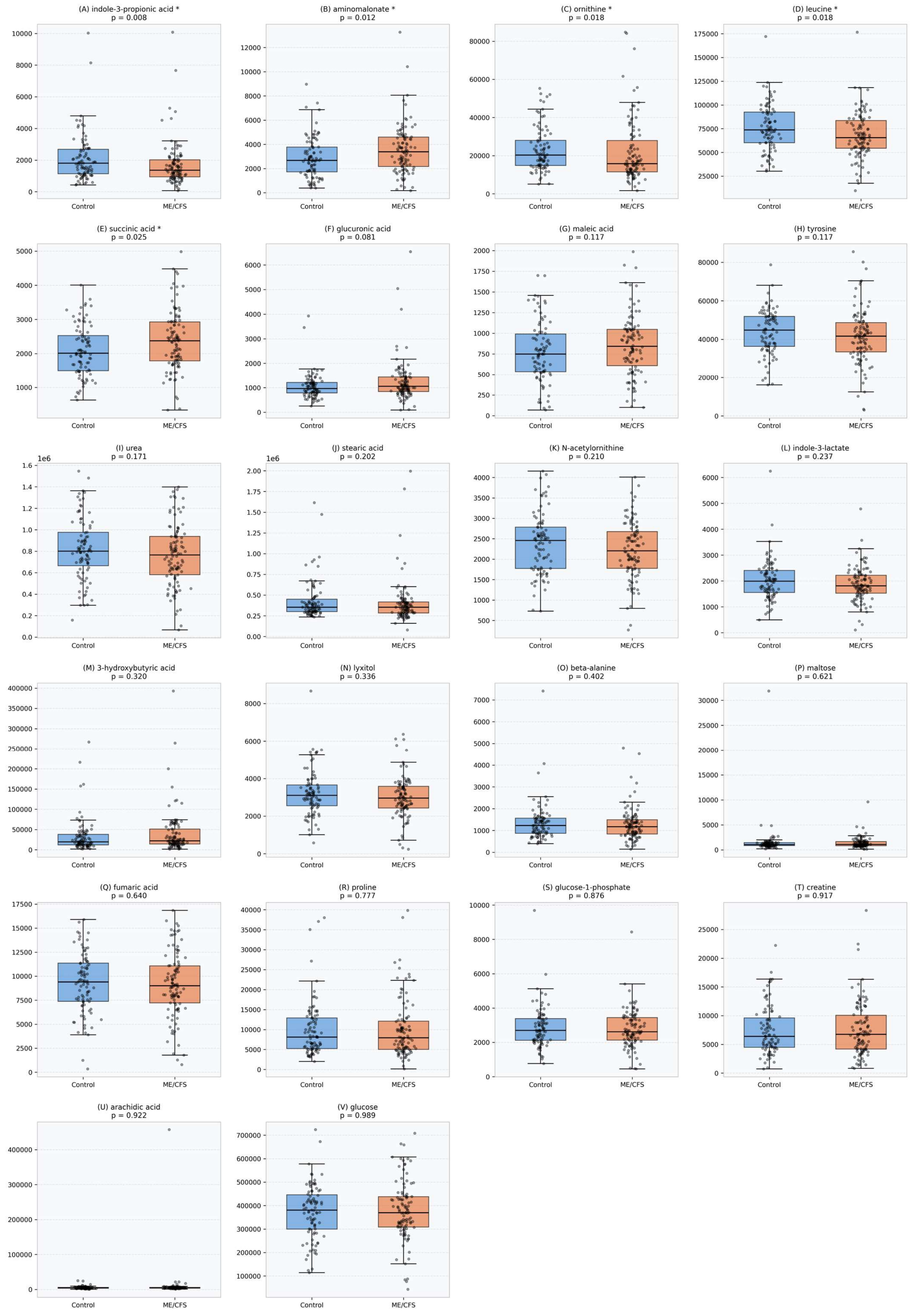
(A) indole-3-propionic acid *
p = 0.008
(B) aminomalonate *
p = 0.012
(C) ornithine *
p = 0.018
(D) leucine *
p = 0.018
(E) succinic acid *
p = 0.025
(F) glucuronic acid
p = 0.081
(G) maleic acid
p = 0.117
(H) tyrosine
p = 0.117
(I) urea
p = 0.171
1e6
(J) stearic acid
p = 0.202
1e6
(K) N-acetylornithine
p = 0.210
(L) indole-3-lactate
p = 0.237
(M) 3-hydroxybutyric acid
p = 0.320
(N) lyxitol
p = 0.336
(O) beta-alanine
p = 0.402
(P) maltose
p = 0.621
(Q) fumaric acid
p = 0.640
(R) proline
p = 0.777
(S) glucose-1-phosphate
p = 0.876
(T) creatine
p = 0.917
(U) arachidic acid
p = 0.922
(V) glucose
p = 0.989
Control
ME/CFS

Figure S1. Case-Control Distributions of Top-Ranked Individual Metabolites

Box plots show the distribution of raw abundance values for healthy controls (blue) and ME/CFS cases (orange) for each of the 22 individual metabolites comprising the top-15 ranked EBM terms (Figure 3A and 3B). Boxes denote the interquartile range (IQR) with the median indicated by the horizontal line; whiskers extend to 1.5× IQR. Individual data points are overlaid with jitter for visualisation. Asterisks denote nominal statistical significance (Mann-Whitney U, $p < 0.05$); panels are ordered by ascending p value.
